# Development of an embryo transfer model to study uterine contributions to pregnancy *in vivo* in mice

**DOI:** 10.1530/RAF-21-0087

**Published:** 2022-01-17

**Authors:** Meaghan J Griffiths, Lauren R Alesi, Amy L Winship, Karla J Hutt

**Affiliations:** 1 Department of Anatomy and Developmental Biology, Development and Stem Cells Program, Biomedicine Discovery Institute, Monash University, Clayton, Victoria, Australia

**Keywords:** endometrial receptivity, pregnancy, ovariectomy, embryo transfer, infertility

## Abstract

**Graphical abstract:**

A mouse model to study uterine specific contributions to pregnancy.

Maternal environmental exposures can exert impacts on the ability of the uterus to sustain healthy pregnancy. To establish an *in vivo* model to study this, we designed an ovariectomized mouse embryo transfer model. The rationale being future studies could expose recipient female mice to variables such as altered diet, drug, temperature, air, or activity exposure among others to define their impacts on the uterine contribution to pregnancy. Ovariectomy ensures the extent of the variable is limited to exploring outcomes on uterine but not ovarian function. Embryo transfer from healthy, unexposed donor mice guarantees that any impacts of the variable are attributed to the maternal uterine but not the embryonic state. Pregnancy outcomes including pregnancy success (number of implantation sites) and viability (number of viable vs resorbing implantation sites) can be investigated. Numerous functional outcomes can be assessed, including developmental competence encompassing decidual, placental, fetal, and vascular morphology and/or function (e.g. measured using Doppler ultrasound, comparisons of fetal growth, or molecular or histological characterization of the decidua, placenta, and fetal tissues).

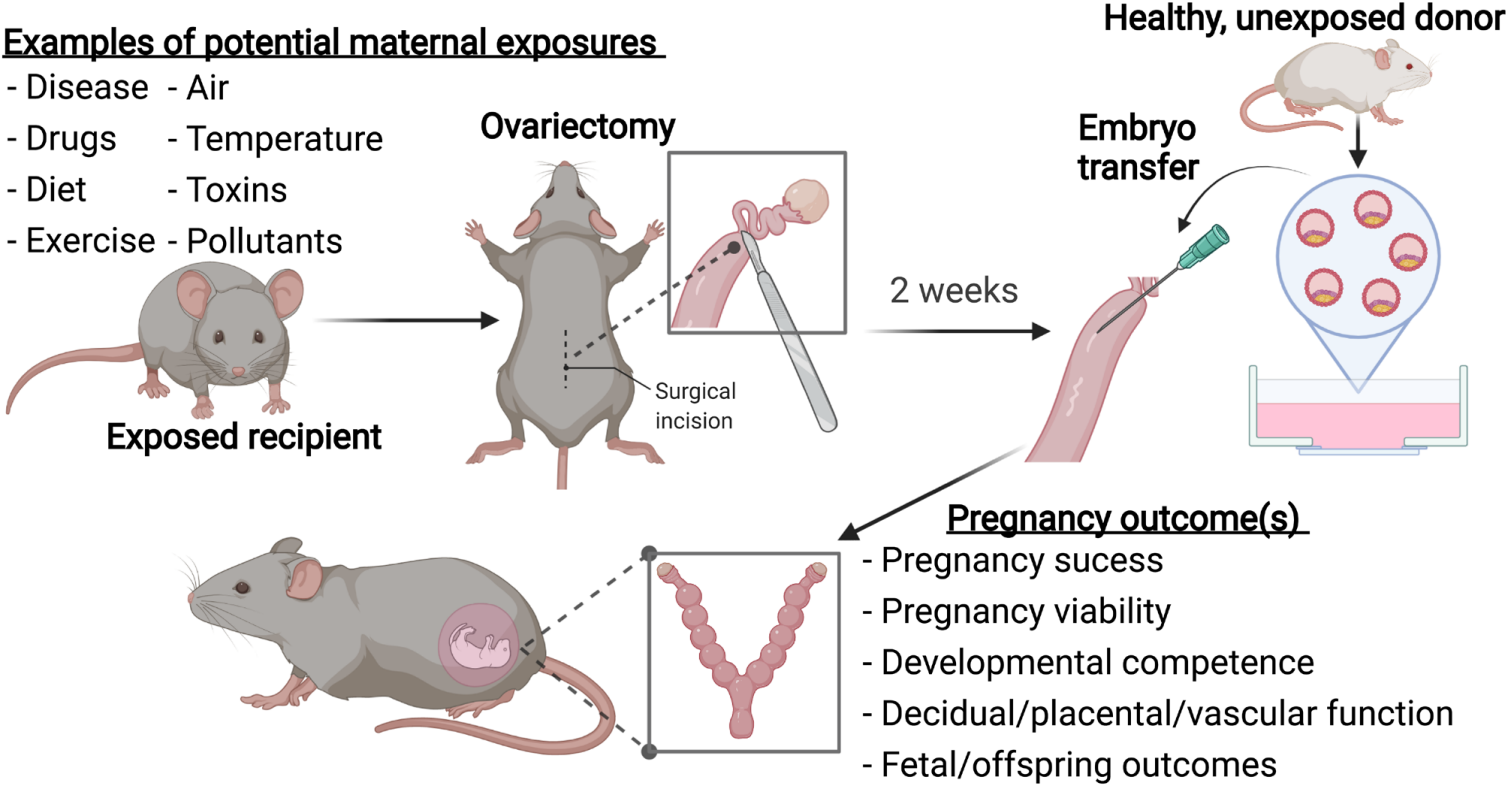

**Lay summary:**

Many pregnancy complications occur because of problems in the womb (uterus), specifically the womb lining. There is a close relationship between the hormone function of the ovaries and the uterus and distinguishing between the way they both impact pregnancy success is difficult in existing studies using animals. Here, we developed a new animal model to utilize in addressing these gaps in our understanding of pregnancy.

## Introduction

Assisted reproductive technologies have rapidly changed the landscape of family planning. However, clinical pregnancy rates following *in vitro* fertilization or intracytoplasmic sperm injection have stagnated in recent years (Norwitz *et al.* 2001, Kupka *et al.* 2014, Ledee *et al.* 2016), indicating many morphologically good-quality embryos fail to implant and establish a pregnancy. This suggests other barriers, including uterine deficiencies, contribute to the limited pregnancy success rates observed clinically (Richter *et al.* 2006, Ruiz-Alonso *et al.* 2013, 2014, Coughlan *et al.* 2014). While informative, human *in vitro* culture models of endometrial receptivity and early implantation processes, including decidualization (Fitzgerald *et al.* 2019, 2021), do not allow for the complete recapitulation of the dynamic processes involved in pregnancy establishment. As such, existing *in vivo* models make it difficult, if not impossible, to study uterine specific contributions to pregnancy success, as distinct from those arising from ovarian impacts.

For the maternal environment to be optimally prepared for pregnancy, the endometrium must be receptive to a viable embryo (Evans *et al.* 2016). Once implantation occurs successfully, the maternal stroma terminally differentiates into decidual cells that secrete progesterone to support the pregnancy until the placenta develops (Evans *et al.* 2016). Deficits at any of these early stages can result in pregnancy loss (Wu *et al.* 2018, Yang *et al.* 2019). In recent years, the endometrial receptivity array was developed to assist women with a delayed or shifted window of receptivity to tailor the timing of embryo transfer (ET) to match any individual woman’s optimal receptivity window (Diaz-Gimeno *et al.* 2011, Patel *et al.* 2019). However, the field still lacks an understanding of the uterine drivers of implantation failure and early pregnancy loss and the development of pregnancy complications that manifest after successful implantation.

Female reproductive health and function are reliant on a delicate balance of ovarian hormones that regulate the uterine menstrual cycle. The ovarian granulosa and theca cells produce and secrete estrogen to regulate the menstrual cycle and ovarian follicle development. When ovarian hormones are not produced, the uterus can still respond to exogenous hormone replacement. As an example, young female cancer patients who have undergone sterilizing treatment can successfully transition through puberty with hormone replacement therapy (Critchley *et al.* 2002, Critchley & Wallace 2005, Sudour *et al.* 2010).

The impact of exogenous insults on the ovary’s endocrine function and fertility are well documented with well-established models to demonstrate ovarian follicle depletion following cancer therapies and exposure to environmental toxins (Winship *et al.* 2018). However, oversights and inherent flaws in the study design of these models can mean that any specific uterine damage inflicted by exogenous insults cannot be accurately differentiated from secondary effects arising from ovarian damage. To overcome this issue, ovariectomized mice are commonly used in studies investigating specific insults to the endometrium (Greaves *et al.* 2014, Fullerton *et al.* 2017).

For pregnancy studies, conditional knockout mice using the Cre-lox recombination system are frequently employed, where a tissue-specific Cre-recombinase excises a gene locus flanked by loxP sites to manipulate gene expression in a time- and tissue-specific manner (Feil *et al.* 2009). Driven by progesterone receptor expression, the PR-Cre (Daikoku *et al.* 2008, Fullerton *et al.* 2017) limits loss-of-function of the target gene to tissues and cells expressing progesterone receptor. An important limitation to this system when considering uterine specific deficits is that the expression profile of progesterone receptor extends beyond the uterus, with other tissues including oviduct, ovary, mammary gland, and the pituitary gland (Daikoku *et al.* 2008) also expressing progesterone receptor. This system also does not allow for the uterine specific impacts of exogenous insults to be studied, highlighting the need for an animal model to specifically investigate exogenous factors on uterine function and allow distinction from ovarian and endocrine influence. ETs where blastocysts are collected from donor mice that remain unexposed to an exogenous treatment provide a well-controlled model for studying uterine specific defects in pregnancy.

Here, we used the common inbred C57BL/6J strain, as well as an F1 cross of C57BL6J and BALB/c, and the outbred strain Swiss to optimize an ET model for studying uterine contributions to pregnancy complications. We aimed to define implantation rates following ET across strains. We determined the extent of developmental progression following ET and exogenous hormone supply in each strain and also assessed uterine artery blood flow in pregnancy across each strain.

## Materials and methods

### Animals

Animals were housed in temperature-controlled high barrier facilities (Monash University Experimental Animal Facility), with food and water available* ad libitum* under a 12 h light:12 h darkness cycle. All procedures were approved by the Monash University Animal Ethics Committee and performed in accordance with the NHMRC Australian Code of Practice for the Care and Use and Animals. Surgical procedures for this study were completed by the Monash Animal Research Platform Reproductive Services Team.

### Ovariectomy of embryo transfer recipients

Based on a previously published methodology (Kondoh *et al.* 2009), 6-week-old female C57BL/6J, Asmu:Swiss, or C57BL/6JxBALB/cJAsmu(F1) were ovariectomized. Females were anaesthetized by isoflurane inhalation (1–5%, v/v in oxygen). Analgesic carprofen was administered subcutaneously (5 mg/kg in saline) and eye lubricant was applied. A small region was shaved on the animals back where a paralumbar skin incision was then made. Blunt dissection was used to separate the skin from the underlying muscle before small incisions were made through the peritoneal muscle wall just under the kidney on each side. The ovaries and oviducts were removed, the peritoneal muscle wall incisions were closed with a double suture knot (Sofsilk Silk sutures GS-832, Covidien), and bupivacaine analgesic was applied topically (0.05% in saline). Skin incisions were closed with Michel clips (Daniels, NS-000242). Female mice were then rested for 2 weeks to allow endogenous hormones to subside.

### Embryo transfer donors

Adult female BALB/cJAsmu were superovulated via injection with PMSG (Folligon, 5 IU) on day (D) 1, then hCG (Chorulon, 5IU) on D3, and then mated with a BALB/cJAsmu stud overnight. Those that had plugged the following morning were then humanely culled by cervical dislocation on D6 (gestational day 2.5) and their uterine horns and oviducts flushed for blastocysts. Blastocysts were cultured overnight in M2 media before transfer to recipient animals the following day.

### Embryo transfers to recipients

On D0, females received 100 ng estradiol (Sigma E8875) and then 2 mg progesterone (Sigma P0130) on D2 in sesame oil. On D3, ETs were performed. Females were anaesthetized via isoflurane inhalation (1–5%, v/v in oxygen) and a small region was shaved on the animals back. Analgesic carprofen (5 mg/kg in saline) was administered subcutaneously and a paralumbar incision made through the s.c. layer. Each uterine horn was exposed at the oviduct end via a small incision into the peritoneal wall for transfer of five blastocysts per horn. Peritoneal incisions were closed using double suture knot (Sofsilk Silk sutures GS-832, Covidien) and then bupivacaine (0.05% in saline) was applied topically. At the time of ET, females also received 2 mg progesterone and 25 ng estradiol subcutaneously. Progesterone (2 mg) was supplemented each day post ET, until collection 10 days post ET. Animals were humanely culled by isoflurane inhalation after conclusion of ultrasound imaging, and then uteri were carefully dissected out and formalin was fixed for further analysis.

### Doppler ultrasonography

At 10 days post ET, recipient females were anaesthetized by isoflurane inhalation (1–5% v/v in oxygen) and then transferred to a heated imaging platform in supine position. Uterine artery was identified and imaged using the Vevo2100 system (VisualSonics). Three consecutive waveform measurements were taken from three separate ultrasound recordings of the uterine artery. Measurements included peak systolic velocity (PSV), end diastolic velocity (EDV), and velocity time interval (VTI). Calculations of pulsatility and resistive indices were then calculated as follows: PI = (PSV − EDV)/VTI and RI = (PSV − EDV)/PSV.

### Histological analysis

Whole implantation sites, dissected embryos, or placentas were formalin fixed for 24 h and then processed and embedded in paraffin and sectioned at 5 μm. Sections were dewaxed in histolene twice and rehydrated through graded ethanols (100–70%) and distilled water prior to relevant staining procedure outlined below. For dehydration and clearing, sections were rapidly moved through graded ethanols (70–100%) and cleared in histolene twice before mounting with DPX and glass cover slips.

### Hematoxylin and eosin

Whole implantation sites, dissected embryos, or placentas slides were submerged in hematoxylin for 10 min, rinsed under running distilled water until clear before being immersion in lithium carbonate for 30 s, and rinsed again prior to dip in acid alcohol and finally eosin for 2 min.

### Periodic acid Schiff (PAS)

Placental sections were placed in periodic acid (Amber Scientific, ChemSupply, Australia) for 10 min, rinsed thoroughly with water for 2 min, placed in Schiff’s reagent (Amber Scientific) for 15 min, and rinsed in water again. Sections were then counterstained with hematoxylin only (as described above).

### Statistics

Statistical analysis was performed using Graphpad Prism 9. Data normality was assessed by Shapiro–Wilk test. Significance was assessed using one-way ANOVA with Tukey’s multiple comparison test for parametric data or Kruskal–Wallis test with Dunn’s multiple comparisons test for non-parametric data. A *P* -value of < 0.05 was considered significant.

## Results

**Figure 1:**
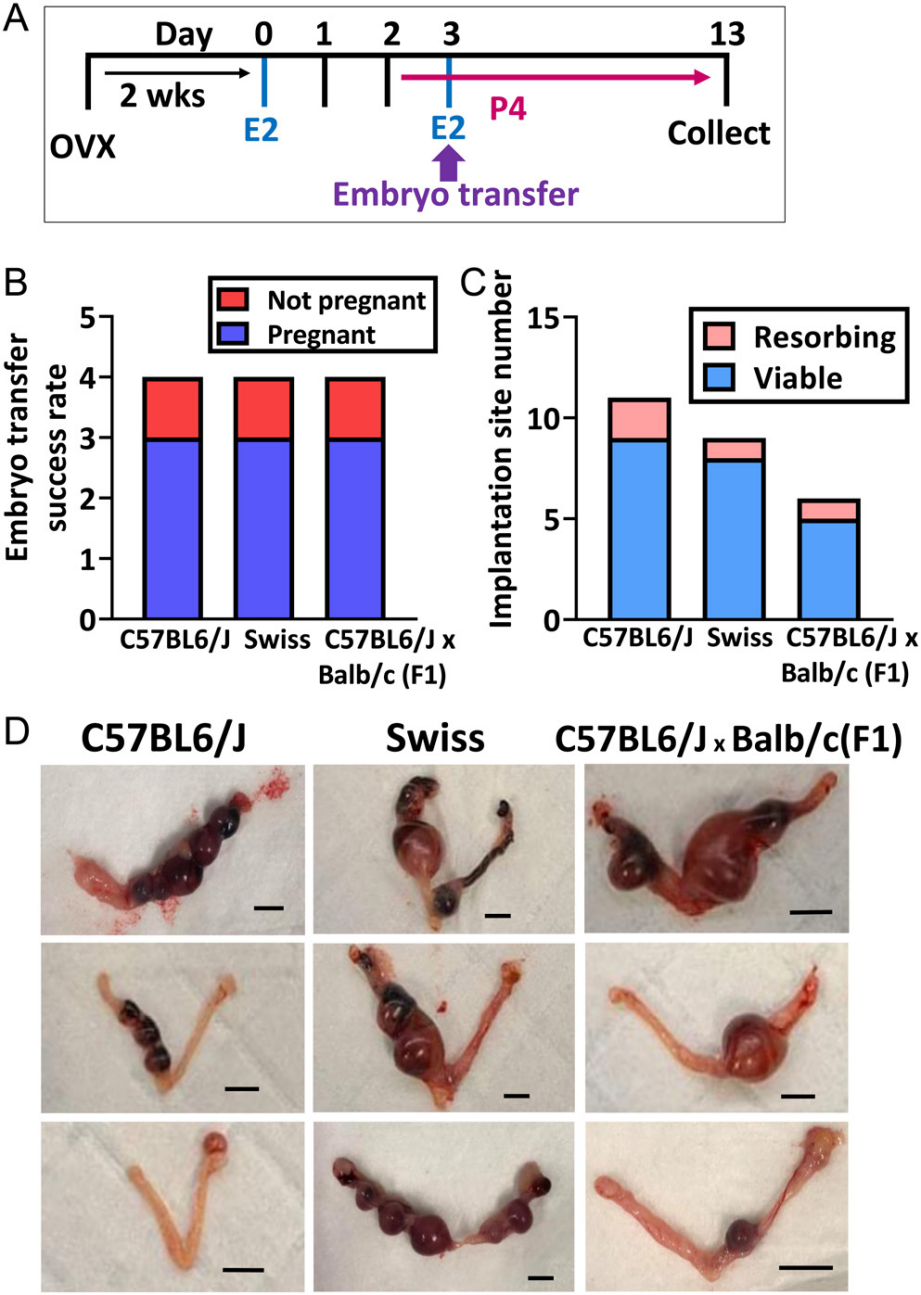
Optimizing an embryo transfer model for studying uterine specific contributions to pregnancy. (A) Young adult female mice were ovariectomized and rested for 2 weeks before hormone priming with estradiol (D0) and progesterone (D2) before embryo transfer surgery on D3. Pregnancy was supported with estradiol (25 ng) at the time of embryo transfer and daily s.c. injections of progesterone (1 mg) from D3 until time of tissue collection. Implantation sites were harvested 10 days post embryo transfer. (B) The embryo transfer success rate shows three out of four mice of each strain were pregnant at D13. (C) Implantation rates per animal were similar. (D) Panels show images of each pregnant uterus from each strain and the variety in developmental stage of each implantation site. Scale bars 5 mm. Data are shown as mean ± s.e.m.; one-way ANOVA with Tukey’s multiple comparisons test; *n*  = 4/group.

To enable distinction between uterine and ovarian contributions to pregnancy success, mice were ovariectomized, hormone primed prior to ET, and whole implantation sites, embryos, and placentas collected 10 days post ET (Fig. 1A). All ET recipient strains C67BL/6J, Swiss, and C56BL/6xBALB/c (F1) received transfer of 8–10 BALB/c donor embryos. C57BL/6J were chosen as they are an extremely commonly used strain as WT and for generating knockout colonies. Swiss:Asmu is an outbred strain useful for determining if there was any difference in the success of our model with more genetic diversity bred into the colony. Finally, we included a mixed background cross of the commonly available C57BL/6J and BALB/c.

Swiss recipient mice had the greatest body weight at the time of collection, despite consistent ages across the recipients (Supplementary Fig. 1, see section on supplementary materials given at the end of this article). ET success rate was 75% for each strain (Fig. 1B). C57BL/6J and Swiss recipient mice had similar numbers of implanted embryos and of the number that were viable was also similar (Fig. 1C and Table 1). C57BL/6J were the least developmentally advanced (Fig. 1D) and had the most resorbing sites (Fig. 1D and Table 1). The outbred Swiss strain were much more developmentally advanced (Fig. 1D) compared to the C67BL/6J females. The F1 females had the smallest number of implantation sites (total implanted and viable implantation sites 1.67 ± 1.16) (Fig. 1D); however, their developmental progression was similar to that of the Swiss females.

**Table 1:** Embryo transfer success. The number of viable implanted embryos, and resorbing non-viable implantation sites for each strain at 10 days post embryo transfer. Data are represented as mean ± s.e.m.

Strain	Transferred	Implanted	Viable	Resorbing
C57BL6/J	10	3.33 ± 1.20	2.67 ± 1.53	0.67 ± 0.57
Asmu:Swiss	10	3.00 ± 1.00	2.67 ± 2.08	0.33 ± 0.57
C57BL6/J × Balb/c(F1)	10	2.00 ± 1.00	1.67 ± 1.16	0.33 ± 0.57

**Figure 2:**
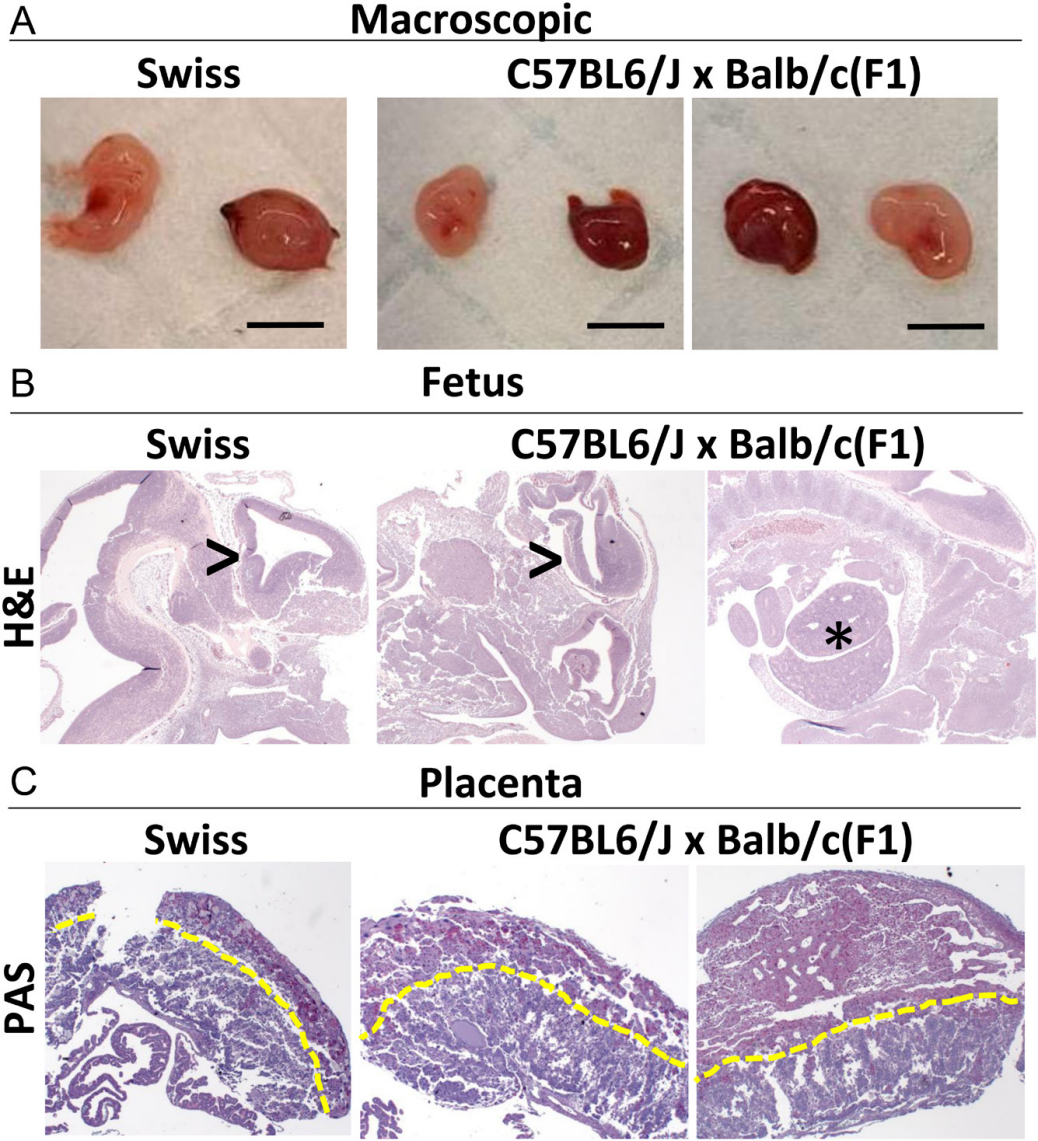
Histological features of fetal and placental tissues from Swiss and F1 mothers. (A) Macroscopic images of dissected implantation sites from Swiss and F1 females, which had the most developmentally advanced implantation sites. (B) Representative cross sections of fetal tissues. Forebrain structures (>) appear less developed in offspring from Swiss mothers compared to the F1 strain. Additionally, development of other organs including the liver (*) are apparent in offspring from F1 mothers. (C) From representative photomicrographs of PAS staining, there were no apparent differences in gross placental morphology between strains. Yellow dotted line separates labyrinth and spongiotrophoblast sections of the placenta. Images taken at 10× objective.

Due to the advanced developmental progression of the Swiss and F1 strains, embryos and placentas were dissected from each of these strains for morphological and histological assessment (Fig. 2A). This was not possible for C57BL/6J implantation sites because they were at an earlier developmental stage. Hence, the placenta and embryo were not yet fully formed.

Developmentally, embryos that developed in Swiss mothers were less advanced compared to embryos from the F1 strain. Figure 2B displays the developing brain structures of an embryo from a Swiss mother, compared to the more advanced F1 strain embryo which displays more developed brain structures and abdominal organs, with the liver observable (Fig. 2B). There were no obvious differences in gross morphology of the placentas between maternal strains with clear distinction between the labyrinth and spongiotrophoblast layers in both strains visualized through PAS staining (Fig. 2C).

**Figure 3:**
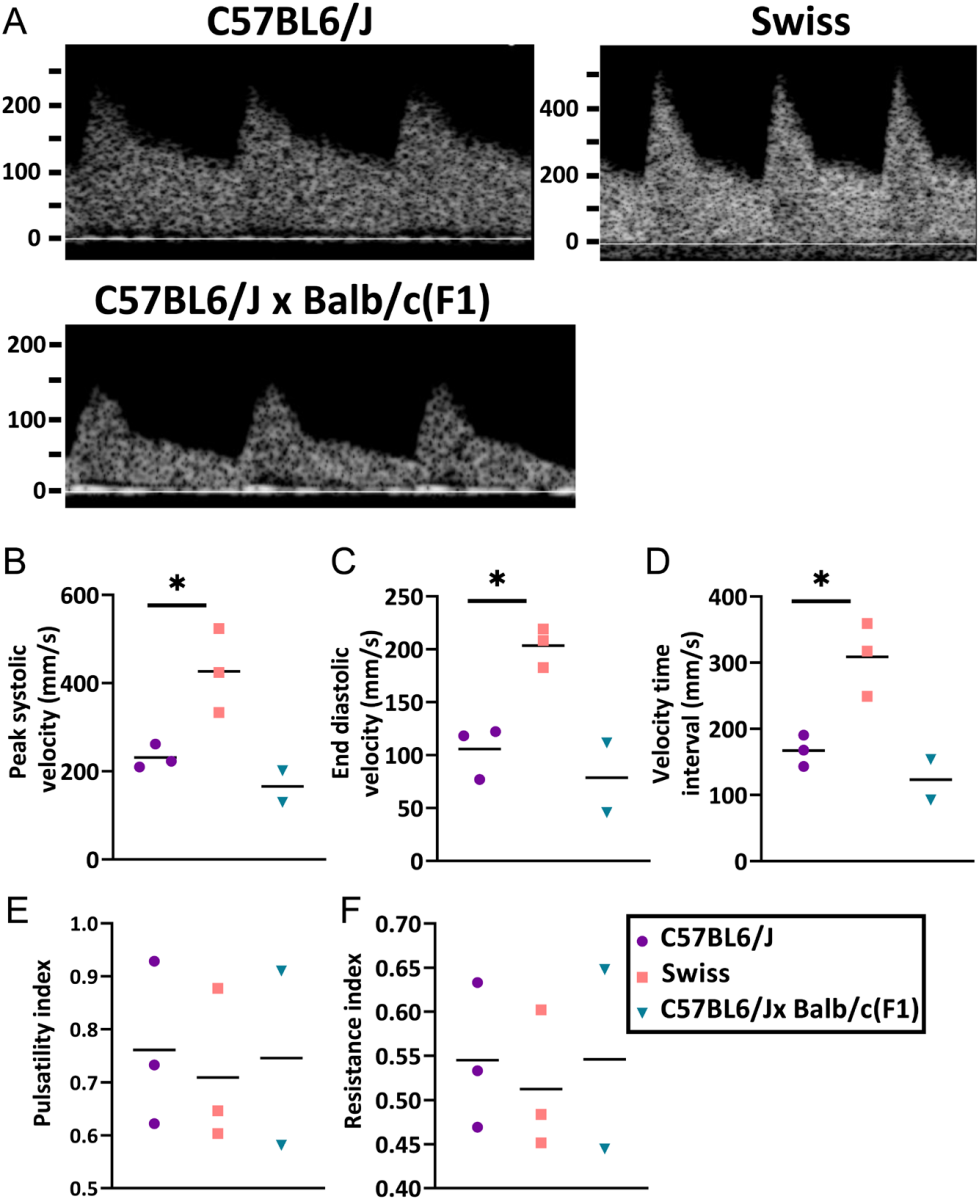
Uterine artery blood flow is most readily detectable by Doppler ultrasound in the outbred Swiss strain. (A) Representative images of uterine artery waveforms. Outbred Swiss have greater peak systolic velocity (B), end diastolic velocity (C), and velocity time interval (D). No changes in pulsatility (E) or resistance (F) indices. Data are shown as mean ± s.e.m.; unpaired *t*-test between C57BL6J and Swiss groups only for (B-F); *n*  = 2–4/group.

Adaptations to maternal blood flow during early pregnancy are critical for ongoing pregnancy success. Doppler ultrasonography waveforms are greatly informative, both in their shape and velocity measurements (Clark *et al.* 2018). Clinically, uterine artery Doppler is used to assess the development of pregnancy complications (Nakatsuka *et al.* 2003, Papageorghiou & Leslie 2007, Ridder *et al.* 2019). Measurements of uterine artery blood flow taken with Doppler ultrasonography revealed significantly higher peak systolic velocity, end diastolic velocity, and velocity time interval in outbred Swiss mice compared to C57BL/6J mice (Fig. 3). While the F1 strain also appeared to have lower velocity blood flow, statistical analysis was not possible due to the small sample size. Pulsatility and resistance indexes were similar for all strains, indicating changes to the velocity of the system did not appear to influence the function of the uterine artery (Fig. 3).

## Discussion

Here, we present a strategy for deciphering damage to the uterus from ovarian or endocrine influence in a mouse model. By ovariectomizing mice and then providing hormone support exogenously alongside ET from healthy donor mice, specific insults to the uterus and their impacts on pregnancy can be determined. We compared this model in three commonly available and widely utilized strains including the inbred C57BL/6J strain, outbred Asmu:Swiss, and the C57BL/6JxBALB/c(F1) strain. Depending on the research interests and biological questions being asked by researchers, each of these strains have their advantages and disadvantages (Table 2); however, the key finding of this study advocates that ET success is possible in each of the recipient strains used.

**Table 2:** Advantages and weaknesses of each strain for use in embryo transfer studies.

Strain	Advantages	Limitations
C57BL6/J	• Multiple implantation sites • Ideal for studies of early implantation	• Slower developmental progression • May require clip repairing or culling post surgery
Swiss	• High-velocity uterine blood flow • Ideal for decidualization and placentation studies	• May require clip repairing or culling post surgery
C57BL6/J × Balb/c(F1)	• Low risk of interventions post surgery • Ideal for decidualization and placentation studies	• Difficult to detect blood flow

For studies interested in the peri-implantation and early implantation periods (Table 2), C57BL/6J may be most useful as they can generate several implantation sites per animal and are readily available. This is reassuring knowledge as an abundance of genetically modified mouse models are maintained on a C57BL/6 background (Bryant 2011, Fontaine & Davis 2016).

Alternatively, for studies focussed instead on decidualization or placentation (Table 2), the Swiss or F1 strain may be more appropriate. Swiss have many advantages including being an outbred strain which introduces immune system and genetic diversity (Martin *et al.* 2017). The Swiss recipient females also had easily detectable and high velocity uterine artery blood flow as determined by Doppler ultrasound making them great candidates for studies focussed on vascular remodeling in pregnancy directly resulting from uterine defects.

In contrast, the F1 strain had much smaller litters, and the uterine artery blood flow was difficult to detect. However, an advantage of the F1 strain is their demeanor and physical condition; they were easy to handle and tolerated surgeries well, with no interventions required to manage the surgical clips. Whereas, both Swiss and C57BL/6J recipients required clip repairs and culling post surgery due to issues with surgical clips. The F1 strain also make good mothers as they are less likely to cannibalize their litters, which the C56BL/6J mothers are known for (Weber *et al.* 2013, Brajon *et al.* 2021).

Until now, it has been difficult to differentiate damage to the uterus from damage to other reproductive organs, even in the commonly used PR Cre-lox system. However, a key limitation to the PR-Cre model lies in the fact PR is expressed in many tissues outside the uterus. While useful for conditional genetic deletions, the PR-Cre system suffers the same shortfalls in limiting researchers’ ability to decipher uterine damage from damage to other organs or tissues that may be having downstream impacts on the uterus.

A limitation in our model for consideration is the influence of seminal fluid on the uterine microenvironment and its impact on pregnancy success (Schjenken & Robertson 2015, 2020). Mating recipient females with vasectomized males is a well-established protocol for inducing pseudo-pregnancy in female mice (Barrette *et al.* 2012, Rhee *et al.* 2016). The literature suggests that seminal plasma exposure is important for successful pregnancy establishment (Schjenken & Robertson 2015) due to its involvement in generating a maternal immune response to promote tolerance to an implanting blastocyst. Despite this, ET was successful in 75% of females. In the future, introducing a vasectomized mating to this model could further improve the pregnancy and implantation success rates.

The use of non-surgical ET (NSET) devices is a well-established method for performing ET surgeries non-invasively. While this device offers a great non-surgical alternative, NSET devices are expensive to purchase commercially and less efficient when made in house. However, the procedure of a NSET could be completed by any trained researcher, while the surgical alterative requires expertise that may not be available to all researchers or institutes.

The findings from this study provide a robust model for studying the specific impacts to the uterus in response to any exogenous insult. By ovariectomizing to remove the confounding factor of any ovarian damage following an external insult and subsequently providing healthy donor embryos from unexposed mice, the specific outcomes on the uterus and on pregnancy following can be elucidated.

## Conclusions

Here, a novel model is described that is designed to differentiate uterine from ovarian influences on pregnancy. Specific impacts to the uterus in response to exposure to exogenous factors can be interrogated using this model, independent of ovarian and endocrine impacts. Examples of exogenous factors include maternal diets, pesticide or herbicide exposure, environmental conditions like smoke or hypoxia exposure, or genotoxic treatments like cancer therapies. This model can also be expanded to investigate exposure to potentially beneficial factors to pregnancy success, with the intention of furthering our understanding of the uterine specific factors that help or hinder pregnancy success.

## Supplementary Material

Supplementary Figure 1. (A) Body weight (g) and (B) age (days) of female recipient mice at D13 of the study, at tissue collection. Mean of data shown ± SEM; one-way ANOVA with Tukey’s multiple comparisons test; n=4/group.

## Declaration of interest

The authors declare that there is no conflict of interest that could be perceived as prejudicing the impartiality of the research reported.

## Funding

This work was made possible through Victorian State Government Operational Infrastructure Support and Australian Government http://dx.doi.org/10.13039/100015539 NHMRC IRIISS. M J G and L R A are supported by an Australian Government http://dx.doi.org/10.13039/100015539 Research Training Program Scholarship and L R A a Monash Graduate Excellence Scholarship. This work was supported by generous funding support of a Monash University http://dx.doi.org/10.13039/501100001779 Faculty of Medicine, Nursing & Health Sciences Platform Access Grant to A L W (PAG18-0343). A L W and K J H are supported by funding from the Australian Research Council http://dx.doi.org/10.13039/501100000923; A L W - DE21010037 and K J H - FT190100265.

## Author contribution statement

M J G, A L W, K J H conceived and designed the study. M J G, L R A performed experiments. M J G analysed data and all authors interpreted the data. M J G and A L W wrote the manuscript. All authors edited the manuscript. A L W or K J H contributed equally to this work.
