# Supplementary figures and images for: Development of an embryo transfer model to study uterine contributions to pregnancy in vivo in mice
Source: Reprod Fertil. 2022 Jan 17;3(1):10–8. doi: 10.1530/RAF-21-0087 (PMC8861889; doi:10.1530/RAF-21-0087)

# Supplementary Figure 1

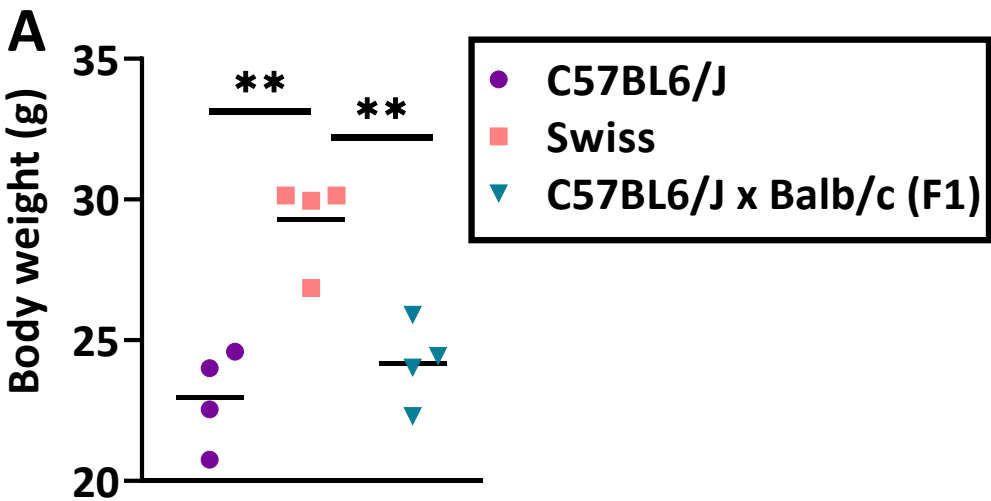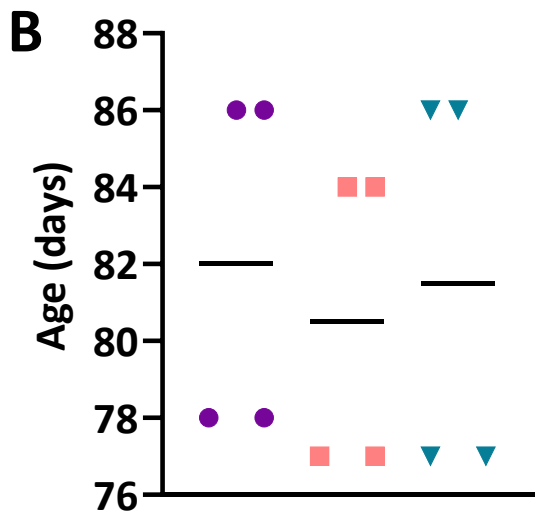

Supplement: Supplementary Figure 1. (A) Body weight (g) and (B) age (days) of female recipient mice at D13 of the study, at tissue collection. Mean of data shown ± SEM; one-way ANOVA with Tukey’s multiple comparisons test; n=4/group. [file supplementary_figure_1.pdf]
